# Research Progress on Surface Damage and Protection Strategies of Armature–Rail Friction Pair Materials for Electromagnetic Rail Launch

**DOI:** 10.3390/ma17020277

**Published:** 2024-01-05

**Authors:** Xing Wang, Pingping Yao, Haibin Zhou, Kunyang Fan, Minwen Deng, Li Kang, Zihao Yuan, Yongqiang Lin

**Affiliations:** 1 State Key Laboratory of Powder Metallurgy, Central South University, Changsha 410083, China; xing357wang@163.com (X.W.); 18874108043@163.com (M.D.); klcxcus@126.com (L.K.); yzh19967803820@163.com (Z.Y.); jxust_lyq@163.com (Y.L.); 2 College of Materials Science and Engineering, Central South University of Forestry and Technology, Changsha 410004, China; zhbtc22@126.com; 3 School of Mechanical Engineering, Chengdu University, Chengdu 610106, China; fankunyang@cdu.edu.cn

**Keywords:** armature–rail friction pair, damages of materials, material properties, surface treatment technology, electromagnetic rail launch

## Abstract

Electromagnetic rail launch technology has attracted increasing attention owing to its advantages in terms of range, firepower, and speed. However, due to electricity-magnetism-heat-force coupling, the surface of the armature–rail friction pair becomes severely damaged, which restricts the development of this technology. A series of studies have been conducted to reduce the damage of the armature–rail friction pair, including an analysis of the damage mechanism and protection strategies. In this study, various types of surface damage were classified into mechanical, electrical, and coupling damages according to their causes. This damage is caused by factors such as mechanical friction, mechanical impact, and electric erosion, either individually or in combination. Then, a detailed investigation of protection strategies for reducing damage is introduced, including material improvement through the use of novel combined deformation and heat treatment processes to achieve high strength and high conductivity, as well as surface treatment technologies such as structural coatings for wear resistance and functional coatings for ablation and melting resistance. Finally, future development prospects of armature–rail friction pair materials are discussed. This study provides a theoretical basis and directions for the development of high-performance materials for the armature–rail friction pair.

## 1. Introduction

**Figure 1:**
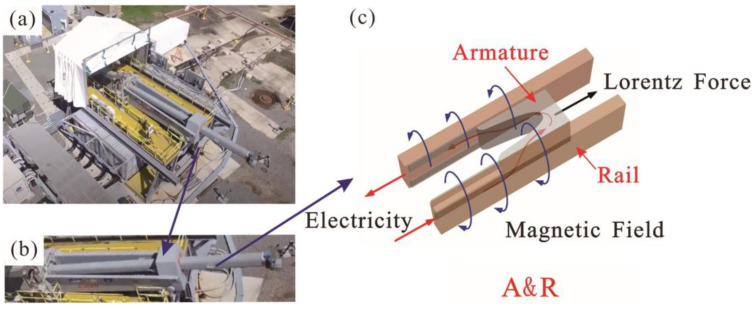
Schematic diagram of electromagnetic rail launcher and A&R. (**a**) Electromagnetic railgun. (**b**) Railgun barrel. (**c**) A&R.

Electromagnetic rail launch is an advanced launch method that uses electromagnetic energy to accelerate a projectile to super-speed. Compared with the traditional launch methods that use mechanical and chemical energy, the launch speed of an electromagnetic rail launch can exceed 2 km/s [1]. With advantages such as considerable firepower input, great range, ample bomb storage, and flexible combat use, electromagnetic rail launch technology has become an essential part of future weapon systems and has been widely studied by science and technology departments worldwide. The electromagnetic rail launcher consists of a high-power supply, two metal rails, and an armature that conducts electricity between the rails to form a conductive loop. During the launch process, the current-carrying armature is driven by Lorentz force generated by a high-intensity magnetic field to a high-speed slide on the rail surface [2]. As a result, good electrical contact is necessary for a successful electromagnetic launch [3]. To achieve this, an interference fit is used between the armature and rails [4,5]. Owing to the high-speed relative motion and friction between the armature and rails, their combination is usually referred to as the armature–rail friction pair (A&R), as shown in Figure 1.

During the launching process, the extreme environment of high electrical current density, high mechanical shock, high temperature rise, and strong strain pose great challenges to the material of the A&R and its contact stability. Because of the interaction within the A&R, a series of complex surface behaviors occur on the surface of the A&R, such as Joule heating, arc electrical behavior [6,7,8], and changes in contact status (solid–arc–solid, solid–liquid–solid, and solid–solid contact). Several types of damage can occur on the A&R surface (friction and wear, deposition, gouge, grooving, transition, the arc ablation of the rail, and melting of the armature) [9,10,11,12,13,14]. These extreme service environments and complex surface behaviors extensively reduce service life and work efficiency, which has become a bottleneck that restricts the development of electromagnetic rail launch technology.

This review aims to summarize the research progress on the surface damage of the A&R surface, focusing on determining the characteristics and formation mechanism, and then introducing protection strategies for these damages.

## 2. Surface Damage of A&R

**Figure 2:**
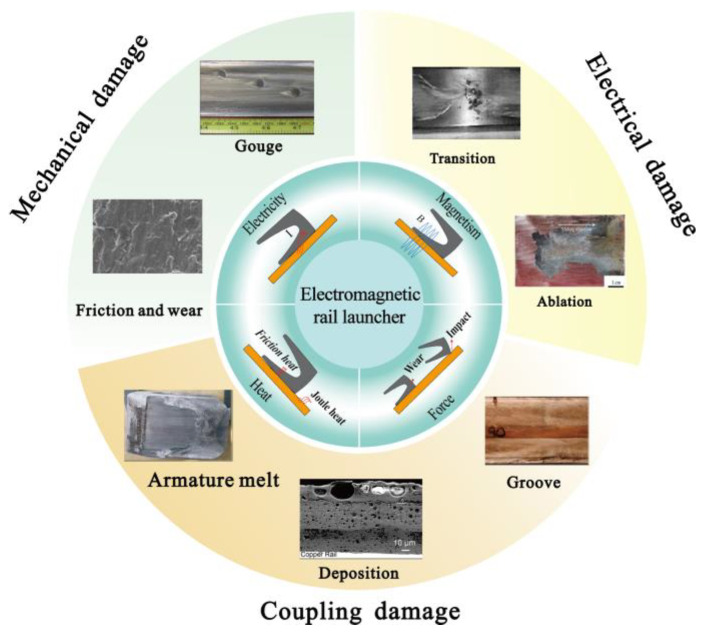
Typical surface damage phenomenon of the A&R.

Due to the coupling of electricity, magnetics, heat, and forces, various types of damage occur on the surface of the A&R. Overall, the damage can be divided into three types, as illustrated in Figure 2. The first type is mechanical damage caused by contact, such as gouging, friction, and wear. The second type is the electrical damage caused by currents and arcs, such as transition and arc ablation. The third type includes deposition, armature melts, and grooves. This type of damage is considered to be caused by the coupling of mechanical and electrical damage.

### 2.1. Mechanical Damage

**Figure 3:**
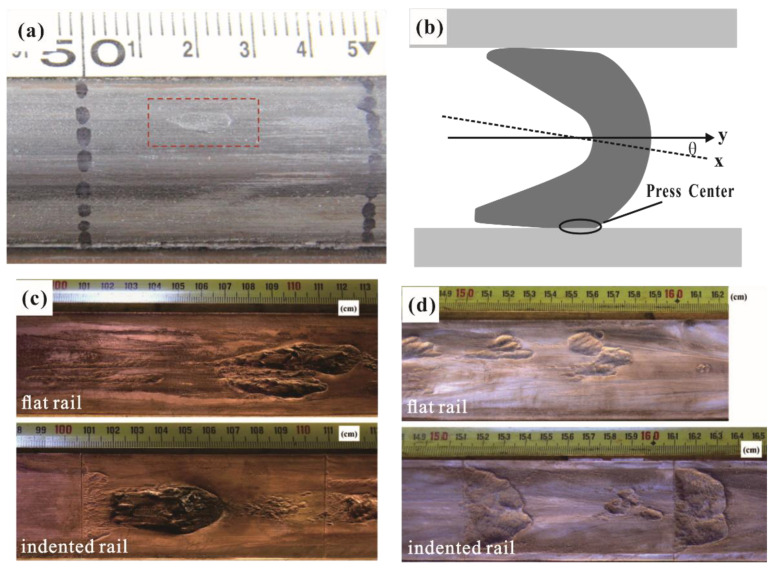
(**a**) Typical teardrop-shaped gouge crater; (**b**) oblique impact model of armature, comparison of gouging on flat and indented rail surfaces at the speed of (**c**) ~1100 m/s and (**d**) ~1450 m/s [15].

A gouge is a typic of mechanical damage to the rail that degrades the contact performance and shortens the service life of an electromagnetic rail launcher [15]. It is widely believed that gouges are caused by the high-speed oblique impact of the armature on the rail. When the strength of the rail material is insufficient to resist the shear force, a teardrop-shaped defect, called a gouge, is produced on the rail, as shown in Figure 3a. Gouges were first found in railguns by Barber and Bauer in 1982 [16]. In addition, the occurrence of gouges on the rail followed a specific pattern. The gouges mainly occurred in the middle and lower regions near the outer edge of the rail. This phenomenon is attributed to the higher contact forces experienced in the middle region of the rail [17]. Most researchers have used simulation and computational methods to study the gouging mechanisms. Lin [18] developed a dynamic model to investigate the mechanism of rail gouging. In this dynamic model, the armature underwent lateral balloting and rotation. With rapid wear and tear at the A&R interface contact, the armature gradually tilted towards one side of the rail, leading to an inclined impact on the guide rail, which may result in a gouge. Furthermore, Wu et al. [19] established a 3D numerical model to simulate and analyze the mechanism and evolution of the gouging phenomenon on a rail. Their findings indicated that surface protrusions on the rail played a significant role in causing gouges. The high-density and high-pressure material flowing on the contact surface could lead to a gouge when the armature is accelerated to high speeds and obliquely extruded into the rail. Watt et al. [15] studied the effect of surface indentations on gouging by carrying out launch tests. They found that the threshold velocity for gouging largely appeared unaffected by the presence of macroscopic surface indentations; however, the shape of the gouges was significantly affected by the indentations. Both the experimental and simulation results showed that the essence of the gouge is the plastic deformation of the rail material. Strengthening materials should be a fundamental method to avoid the generation of gouges.

**Figure 4:**
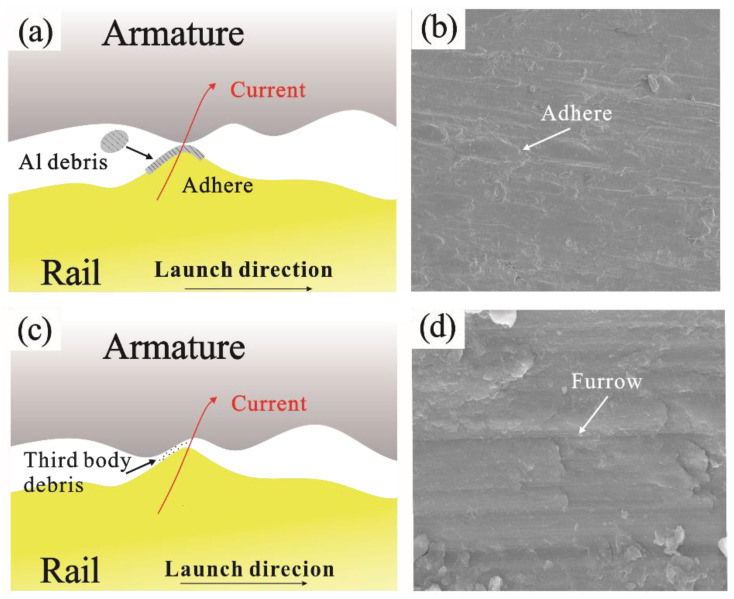
Mechanism and morphology of mechanical wear: (**a**) mechanism of adhesive wear, (**b**) morphology of adhesive wear, (**c**) mechanism of ploughing wear, (**d**) morphology of ploughing wear.

Owing to the preload and high relative sliding speed, serious friction and wear occurs between the A&R during the launch process, as shown in Figure 4. Surface wear also weakens the contact state of the A&R. Brown et al. [20] used a mesoscale friction tester to measure the friction coefficient of the A&R contact interface and found that the friction coefficient decreased by approximately 50% under the action of a current. During the emission process, the wear of the armature was typically more severe than that of the rail. In addition, considerable efforts have been made to investigate the wear of the armatures. Stefani and Parker [21] measured the wear quantity of an armature through an aluminum alloy armature wear test experiment and established an initial wear model for the armature. Wu et al. [22] constructed mathematical models of wear, nonlinear electrical contact, and aerodynamic resistance, and obtained the relationship between the armature stress, wear rate, and time. The results showed that the armature wear mainly occurred at low speeds, particularly within 1 m of the breach. Gao et al. [4] established a 3D simulation model to study the wear characteristics of the A&R under the action of an interference fit and Lorentz force. The maximum contact pressure is distributed on both sides of the armature tail. Furthermore, the location of the wear concentration and the interference play a significant role in determining the wear volume, preferably between 0.2 and 0.25 mm. Ren et al. [23] utilized a finite element simulation to predict wear. Their results revealed that the wear of the armature was influenced by the wear coefficient. When the friction coefficient increased from 1 × 10^−6^ to 1 × 10^−3^, the wear of the armature increased by approximately 190 times. Therefore, to reduce the degree of wear between the armature and the guide rail, it is essential to reduce the friction coefficient by improving the lubrication conditions as much as possible.

Based on the aforementioned research results, the mechanical damage to the A&R surface can be attributed to the interaction forces. When the inherent strength of a material cannot resist these interaction forces, two types of mechanical damage occur on the surface. Therefore, in addition to optimizing the structure of the A&R, enhancing the inherent strength of the material and improving the interface lubrication characteristics between the A&R are effective approaches for reducing the mechanical damage to them.

### 2.2. Electrical Damage

Electrical damage is the phenomenon of a solid contact transforming into an arced contact. When plastic deformation occurs on the A&R and the armature softens at high temperatures, there is a clearance between the A&R, leading to plasma and arc formation. Common forms of electrical damage include transition and ablation.

**Figure 5:**
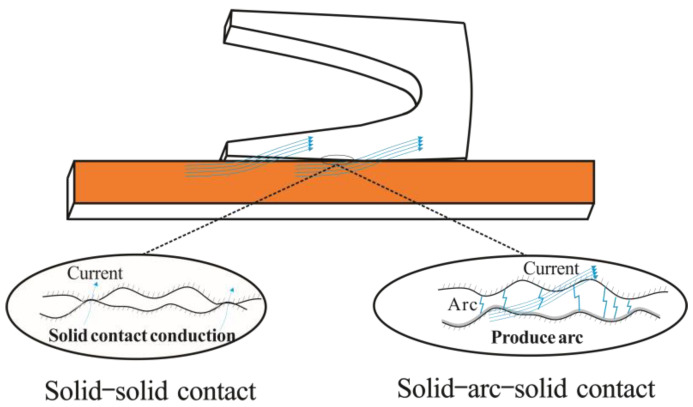
A schematic diagram of the transition.

When the electrical contact changes from a perfect solid contact to an arc contact, a transition appears between the A&R [24], as shown in Figure 5. Currently, there are two widely recognized transition mechanisms [25], the “melt-wave” and the “electrodynamic” mechanisms. The “melt-wave” refers to the movement of the melting layer from the near part to the front part of the contact surface caused by the velocity skin effect. When the wave penetrates the contact surface, there is a gap between the A&R and the transition occurs. Due to the difficulty of experimental observation, the “melt-wave” mechanism has been mainly researched through numerical analysis research methods [26,27,28]. The changes in mechanical properties and electrical contact characteristics caused by varying pulse currents belong to the “electrodynamic” mechanism and are mainly caused by reverse Lorentz force during the current decrease stage [29,30,31]. Some researchers believe that the transition is a complex process, caused by multiple factors. Barber et al. [25] explained in detail the degree to which factors such as preload and electromagnetic contact force, armature strength and current-carrying capacity, the velocity skin effect, inductance, the magnetic giant effect, and wear affect transition. Tang et al. [32] established coupled 3D models of the electromagnetism, temperature, and structure to study the transition mechanism in an electromagnetic launch. They found that the melting wave and electromagnetic force lead to the occurrence of a transition and that the two are inextricably linked.

This transition has many disadvantages, of which surface ablation is the most common. From the initial position to the muzzle end, initial ablation, planning ablation, twisting ablation, and muzzle arc ablation sequentially occur on the rail. Zhang et al. [33] conducted launch experiments under the conditions of a launch current of ~800 kA, more than 100 launches, and an armature mass of 140 g. They observed the damage at the initial position of the rail and analyzed the ablation problems caused by insufficient initial contact pressure, large rail spacing, and insufficient rail strength. In addition, damage caused the muzzle velocity to decrease by 200 m/s and the launch efficiency to decrease by nearly 5% with the same launch conditions. The planning ablation of the rail was led by concentrated pressure, and current density resulted from too small of an area of the A&R contact [34]. When the contact pressure reached the critical strength of the rail, plastic deformation, such as a planning pit, occurred on the rail surface. Electrical current is concentrated in the planning area, leading to the generation of electric arcs, which cause planning ablation phenomena [35]. Twisting ablation is generated by the loss of contact between the A&R caused by the twisting of the rail during the launching process. The two rails are typically considered parallel and symmetric. However, owing to the influence of the structural deformation of the barrel, it often leads to nonideal A&R matching, such as space curving or twisted rails [36]. Muzzle arc ablation refers to the phenomenon of ablation by arcing owing to the breakdown of air by the muzzle voltage when the armature comes out of the bare. Cai et al. [37] analyzed the factors contributing to the muzzle arc formation and performed numerical simulations. The results indicated that the temperature was the highest, the electromagnetic field was the strongest, and the airflow was the most intense when the muzzle arc was formed.

Overall, the transition and ablation phenomena were caused by changes in the contact between the A&R. Therefore, maintaining a stable contact and enhancing the materials resistance to ablation are effective ways to mitigate electrical damage in the A&R.

### 2.3. Coupling Damage

In extremely complex environments, certain forms of damage are not only attributed to a single factor but also arise from the interaction of multiple factors. For instance, friction and Joule heating cause armature melting and deposition, and the grooves are formed owing to thermal stress and aluminum liquid erosion.

**Figure 6:**
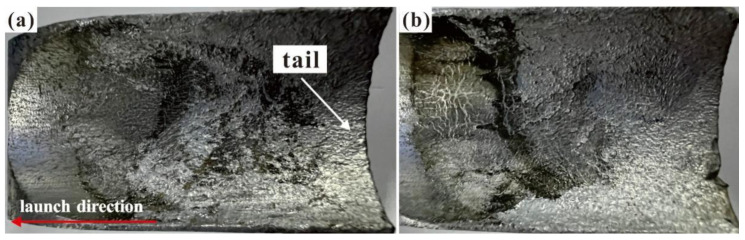
Photographs of recovered armatures: (**a**) positive electrode; (**b**) negative electrode.

For armature melting (as shown in Figure 6), Xia et al. [38] used a payload-separation method to keep the recovered armatures intact. The experimental conditions included an armature and payload mass of 395.05 g, with a peak current ranging from 249.40 to 403.40 kA. They believed that Joule heating was predominant in the melting process. Because the experimental methods are difficult to use when analyzing the effects of multiple factors on the state of contact during launch, numerical simulation is another important research method. Zhang et al. [39] found that a typical butterfly-shaped failure interface appeared in the recovered armature and used the 3D finite method to explain that the non-uniform distribution of current density, heat flux, and Lorentz force density, which was caused by the pulse current, were the main causes of the butterfly-shaped failure interface. Li et al. [40] developed a thermoelastic magnetohydrodynamic model that analyzed the effects of the supply current waveform, armature tail length, and angle on the melting rate of the armature surface as well as the minimum liquid metal film between the contact surfaces. Additionally, some studies have shown that, owing to the current skin effect and tail deformation of the armature caused by stress and structural characteristics, the current is more likely to be concentrated in the armature tail, causing the armature to start melting in the tail [41].

**Figure 7:**
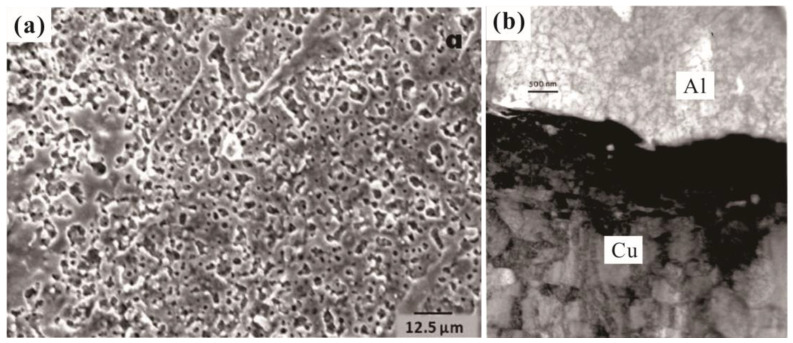
Surface and interface structure of the deposition layer. (**a**) Porous Al deposition; (**b**) TEM bright-field image of interfacial Al/Cu region [46].

In comparison with other forms of damage, the deposition covers almost the entire surface of the rail and occurs throughout the launch process. The formation of the deposition layer is primarily attributed to the transfer of material from the armature owing to friction, melting, and splattering caused by the accumulation of heat [42]. The surface deposition layer on the rail exhibits a variety of typical structures, such as multilayer structures, peeling structures, pores, and surface cracks [10,43,44]. In addition, the deposition exhibited space–time distribution characteristics, with different characteristics observed for varying numbers of shots along the lateral and radial directions of the rail [45]. The deposition layer not only improves the surface roughness of the rail and reduces the electrical contact performance between the A&R, but also creates a solid-state flow regime by dynamic recrystallization at the aluminum/copper interface, which also leads to the erosion-product deposition and damage of the rail, as shown in Figure 7 [46].

**Figure 8:**
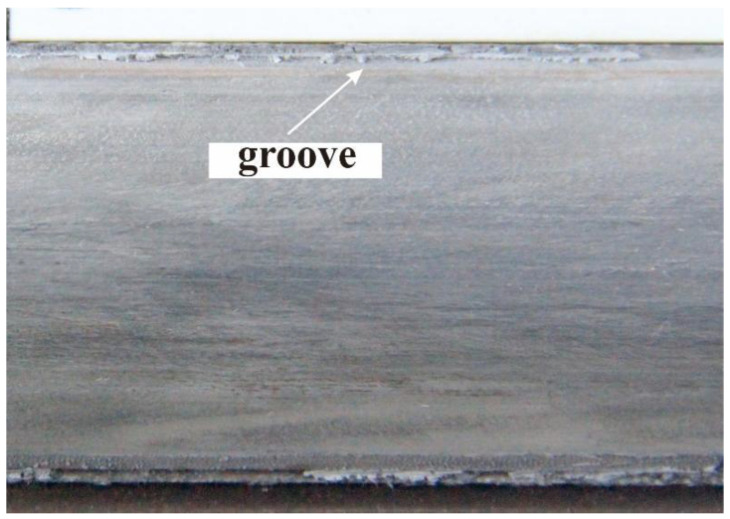
Typical morphology of grooves on the rail surface.

Grooves are a common type of damage to the rail surface that occur after several launches. Grooves typically start with sharp indentations and gradually diffuse, as shown in Figure 8. Grooves are located at the part of the rail exposed to the highest currents and longest armature dwell times and are concentrated in the rail area near the insulator, coinciding with the location of the armature where the damage is the greatest [47]. The formation of grooves is generally attributed to the cumulative effects of thermal softening of the rail material and erosion caused by liquid aluminum from the molten armature [48]. Gee and Persad [49] proposed that this was a plastic deformation phenomenon caused by the chemical action of the rail and molten aluminum. Hsieh [50] used EMAP3D calculations to demonstrate that groove formation was caused by the material softening due to high local temperatures and material yielding. Cote [51] proposed the clamping or shrinkage effects of magnetic pressure on a liquid metal as the cause of groove erosion. The simulation results obtained by Geng et al. [52] indicated that electrical explosions were the primary cause of rail groove formation. The explosion blows off the rail surface, similar to the electric-arc-cutting conductor technique, leaving craters on the rail surface.

### 2.4. Summary of Surface Damage

From the above summary, it can be inferred that the three types of damage to the A&R surface primarily resulted from a series of complex reactions caused by changes in the stress and contact conditions between the A&R during the launch process. Therefore, to mitigate these damages, apart from modifying the structure of the A&R to adjust its stress and contact conditions, enhancing the intrinsic resistance of the material to damage and surface treatment are fundamental and effective approaches for ameliorating the surface damage between the A&R.

## 3. Protection Strategies for Surface Damage

As the demand for a high-speed electromagnetic rail launcher continues to increase, both of the A&R materials face significant challenges. In recent years, Cooper-based rails, such as those made from electrolytic tough pitch copper and oxygen-free high-conductivity copper [53,54,55], and armatures made from Al alloys such as 6061 and 7075 Al [56,57], have been used. Although the materials used for the A&R exhibit high performance, they still fall short of meeting the demand for prolonged lifespans and enhanced reliability in electromagnetic rail launch systems. Thus, there is still a long way to go before A&R materials can be further refined. As a current-carrying friction pair, the contact state and martial strength of the A&R are critical factors in protection strategies for surface damage, which can be achieved by adjusting the structure and pairing, improving the material, and the use of surface treatment technology. Structure and pairing adjustments can improve the distribution of heat and current, avoid contact failure between the A&R caused by high-temperature softening and the deformation of materials, reduce the concentration of current density, and increase the velocity threshold for the transition [58,59,60,61]. Although the adjustment of the structure and pairing can be effective for transition and ablation because the material is the basis of the engineering equipment, the most fundamental guarantee for the long life of an electromagnetic rail launch system is to improve the damage resistance of the A&R materials through material improvement and surface treatment technology.

### 3.1. Material Improvement

Mechanical damage, such as gouging, friction, and wear, is caused by the plastic deformation of materials. This type of damage is attributed to the inability of the material to withstand interaction forces. Enhancing the material strength of the A&R is an effective strategy for addressing this issue. In Copper-based materials, methods of strengthening include alloying techniques such as solid-solution strengthening, precipitation strengthening, grain refinement, and deformation strengthening, as well as composite methods involving the addition of second-phase particles, whiskers, or fibers. Although these approaches can enhance the strength of the matrix material, they may sacrifice its electrical conductivity.

**Figure 9:**
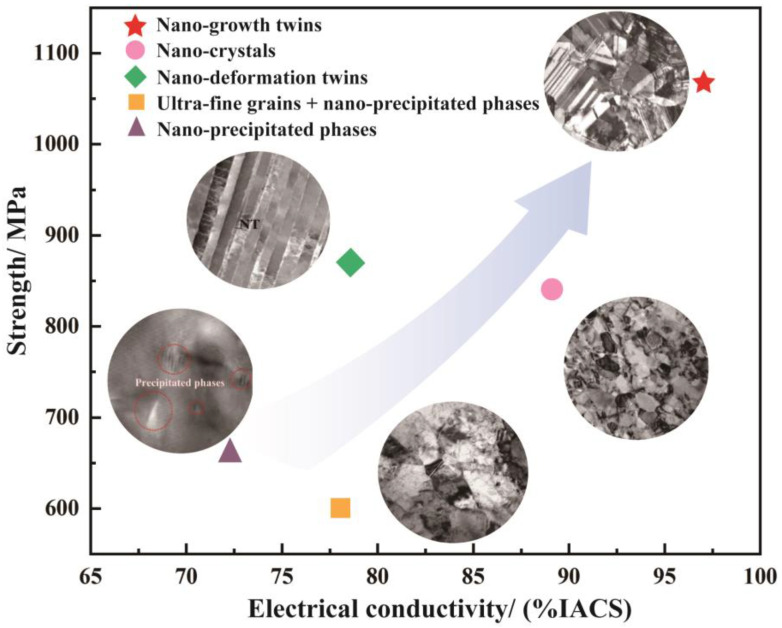
Relationships between typical microstructures and properties of copper alloys with high strength and conductivity [70,71,72,73,74].

The strength and conductivity of Cooper-based materials are influenced by their organization and microstructure, including the precipitated phase, grain boundary, twin boundary, and solute atoms. In terms of strength, the precipitated phase, grain boundary [62], twin boundary [63], and solute atoms [64] hinder the dislocation movement, resulting in a strengthening effect. While, for the conductivity, the total resistivity of the metal can be expressed by the following equation [65]:

$$\rho_{total}=\rho_{0}+\Delta \rho_{dis}+\Delta \rho_{GB}+\Delta \rho_{TB}+\Delta \rho_{SS}+\Delta \rho_{P},$$

where $\rho_{0}$ is the resistivity of pure copper; and $\Delta \rho_{dis}$, $\Delta \rho_{GB}$, $\Delta \rho_{TB}$, $\Delta \rho_{SS}$, and $\Delta \rho_{P}$ are the specific resistance conducted by the grain boundary, dislocation, twin boundary, solute atoms, and precipitated phase, respectively. Grain boundary, dislocation, and twin boundary cause lattice distortion, and impurities are added to the alloy by solute atoms, aggravating the scattering of free electrons and seriously damaging conductivity. For example, Sun et al. [66] added Co and Si to the Cu-Cr alloy, and the results revealed that the addition of Co and Si led to a decrease in electrical conductivity by approximately half. Among the influencing factors, solute atoms have the greatest influence on conductivity compared with the precipitated phases, twin grain boundaries, grain boundaries, and dislocations [64,67,68,69]. Therefore, the typical microstructures of copper alloys with high strength and conductivity include nano-precipitated phases [70], ultra-fine grains and nano-precipitated phases [71], nano-crystals [72], nano-growth twins [73], and nano-deformation twins [74]. The relationships between typical microstructures and properties are shown in Figure 9.

**Figure 10:**
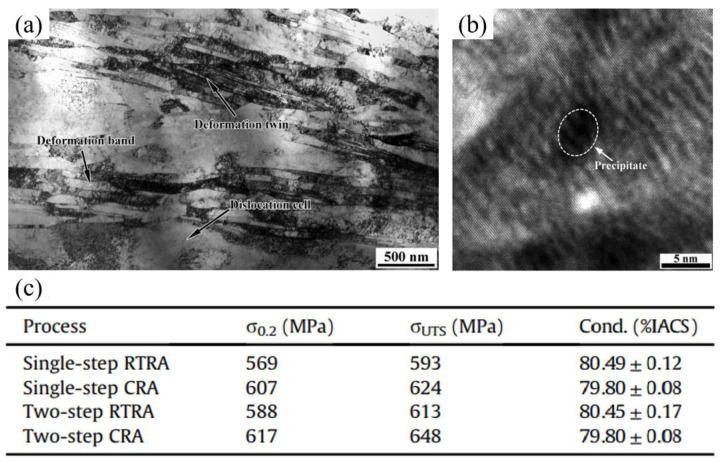
Microstructures and properties of Cu-1Cr-0.1Zr (mass fraction) alloys subjected to two-step CRA. (**a**) Bright-field TEM and (**b**) HR-TEM images; (**c**) table of mechanical properties and electrical conductivity [76].

The CuCrZr alloy, which represents a high-strength and high-electrical-conductivity alloy system, is one of the preferred choices for rail materials owing to its excellent performance. To further enhance the strength and electrical conductivity of CuCrZr alloys, researchers have extensively investigated process optimization and strengthening mechanisms to adjust typical microstructures during their preparation. Li et al. [75] prepared CuCrZr alloys with high strengths and electrical conductivity through aging and cryorolling. The prepared material exhibited a tensile strength of 712 MPa (a 44.72% increase compared to the aged samples), while maintaining a conductivity level of 70.2%. Li et al. [76] performed a two-step cryorolling and aging process (CRA) on the Cu-1Cr-0.1Zr (mass fraction) alloy, and a desired balance between the high tensile strength (648 MPa) and electrical conductivity (79.80% IACS) was achieved due to the coexistence of refined deformation bands, nanoscale deformation twins, and nanoprecipitates (Figure 10). Sun et al. [77] employed the dynamic plastic deformation method at liquid-nitrogen temperature to fabricate block-like CuCrZr nanocomposites consisting of nanotwins and nanograins. The CuCrZr nanocomposite subjected to a single-step deformation and no aging treatment exhibited a tensile strength of up to 700 MPa and an electrical conductivity of 78.5%. Kulczyk et al. [78] carried out experiments on CuCrZr alloy, including severe plastic deformation (SPD), such as hydrostatic extrusion (HE), equal-channel angular pressing (ECAP), and a combination of these processes, all followed by a precipitation hardening stage (solution treatment before the deformation and post-deformation aging). The alloy obtained a high ultimate tensile strength (UTS = 630 MPa) and a high electrical conductivity of 78% IACS.

In addition to the aforementioned results, numerous researchers have achieved simultaneous improvements in strength and conductivity by combining various processes (Table 1). Strengthening processes involve achieving a balance between crystal size, precipitation, and twinning through fabrication techniques. This allows for the attainment of high strength and conductivity in the material.

The high strength of the rail material determines its service life, and its conductivity ensures launch accuracy and efficiency. Both are indispensable for the advancement of electromagnetic rail launch technology. From the existing research results, the process to achieve high strength and high conductivity in copper alloys has the characteristics of combining heat treatment with an ultra-low-temperature dynamic plastic deformation or multi-step deformation process, so that there are nano-scale grains, nano-precipitated phases, or nano-twins in the alloy. However, copper alloys with high strength and conductivity still cannot meet the current development requirements. Therefore, new material systems and processing technologies are the primary focus in the continued development of rail materials.

### 3.2. Surface Treatment Technology

Mechanical, electrical, and coupling damage to the A&R pose a great challenge for the material. To prevent this damage from causing the launch to fail, the electromagnetic rail launcher requires the use of materials with a much higher level of thermomechanical stability. In addition to the material improvement, surface treatment technologies, such as structural coatings for wear resistance and functional coatings for ablation and melt resistance, can also play a role in preventing damage.

#### 3.2.1. Structural Coating for Wear Resistance

**Figure 11:**
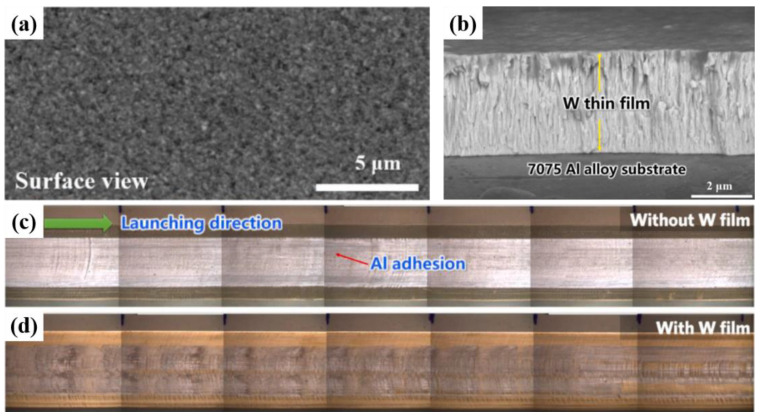
Image of the W coating and Cu rail surface after one shot with and without a W film on the Al alloy armature. (**a**) Surface and (**b**) cross-section image of W coating. (**c**,**d**) Pictures of wear damage and Al adhesion on Cu rail after one shot with and without the W film on the Al alloy armature [82].

When the A&R slides, wear phonemes such furrows and Al adhesion, occur on the surface. Increasing the surface strength of the friction pair and improving the lubrication of the friction interface can reduce the degree of wear of the A&R. Watt et al. [81] pre-coated an Al layer on rails. They conducted practical launch validation experiments with a peak current of ~1200 kA and a maximum muzzle voltage of 700 V, with a ∼140 g lightweight projectile. The result showed that this pre-coating significantly delayed the occurrence of plowing. Lu et al. [82] achieved a significant reduction in the interface energy between the A&R by depositing a W layer on the armature surface using magnetron sputtering, which substantially alleviated the adhesion and mechanical damage on the rail surface (Figure 11). Siopis et al. [83] performed a systematic investigation using the Ashby method to select a rail material that would maximize the magnetic energy for performance, durability, and economic viability. Their results suggested that a hybrid rail material with an electrically conductive substrate and a damage-resistant surface layer consisting of tungsten, chromium, nickel, or tantalum could accomplish these two goals.

#### 3.2.2. Functional Coating for Ablation and Melt Resistance

The results of theoretical calculations have shown that the critical transition speed of pure copper rails was less than 0.5 km/s [84], which means that the arc is inevitable for the copper rail system when the launch speed exceeds 2 km/s. Although copper alloys have outstanding strength and conductivity, their resistance to thermal erosion remains insufficient. The application of an appropriate coating to a rail to enhance its thermal resistance and maintain excellent electrical and thermal conductivities is the primary focus. In addition, owing to the low melting temperature of the Al alloy, a large amount of heat accumulated at the friction interface leads to the melting of the armature surface. Coating the armature surface can improve the lubrication condition of the friction interface of the A&R, which can effectively alleviate the melting of the armature and reduce the generation of the deposition layer to a certain extent.

**Figure 12:**
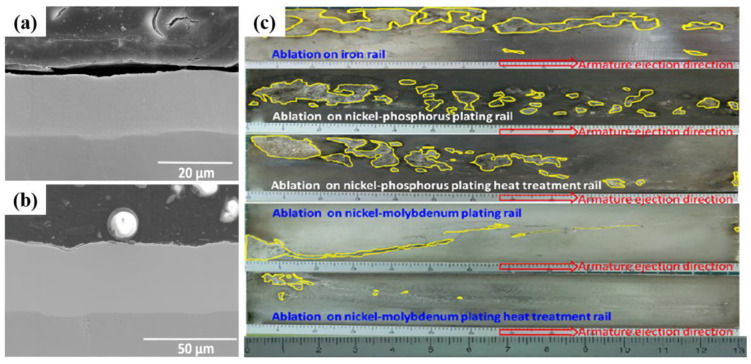
Coating and surface ablation morphology. (**a**) Ni-P Coating, (**b**) Ni-Mo coating, and (**c**) surface ablation morphology of different rails with different coatings. The yellow line area in the figure corresponds to the ablation area of the rail [87].

The coating material for the ablation resistance of rails must have a high melting point, conductivity, wear resistance, and interface compatibility with the rail substrate material. Harding et al. [85] in 1986 used chemical vapor deposition to prepare tungsten and tungsten-rhenium alloy coatings on copper rails. The results showed that the rail performance was significantly better than that of the bare copper rails, and the arc ablation resistance was high. Colombo et al. [86] reported that the wear and spark erosion of copper rails were reduced after the rails were coated with TaN and TiN using Plasma Source Ion Implantation and Ion-Beam-Enhanced Deposition. Hsu et al. [87] applied nickel–phosphorus and nickel–molybdenum coatings to rails and conducted launch experiments with a lightweight armature (27 g). The results showed that the annealed Ni-Mo coating exhibited the best electrical ablation wear resistance (Figure 12). Liu et al. [88,89] used a supersonic plasma spraying technique to prepare Mo-based coatings on copper rail surfaces. This coating exhibited high hardness and excellent ablation resistance.

For the armature surface coating, Zhou et al. [90] performed numerical simulations of armatures with various types of coatings. The results showed that the coating on the rail surface could improve the degree of the concentration of the current density on the contact surface between the A&R, and the maximum temperature was significantly reduced, which could reduce the melting degree of the armature. Lubrication coatings for armatures primarily contain polytetrafluoroethylene (PTFE), graphene, and low-melting metals and alloys. Singer et al. [91] selected PTFE as the coating material to lubricate the contact surface, and their results showed that PTFE not only increased the launching speed, but also effectively reduced armature damage and A&R contact ablation. This plays an important role in improving the launch performance of the system. However, because PTFE is an insulator, the contact resistance and other properties were not considered in the experiment. Du et al. [92] coated a layer of graphene on an armature and conducted emission tests to study the effect of the graphene coating on the surface melting of the armature. After recovering the armature, it was found that the melting area after coating was smaller. Hsieh et al. [93] added low-melting-point alloys such as bismuth, tin, and indium to the surface of an aluminum armature to form boundary lubrication and conducted an experimental study on the melting wear characteristics of the materials on the surface of the armature controlled by the type of coating. The results showed that the armature exit speed slightly increased. However, the experimental speed was only 360 m/s, and a high-speed launch test has not been verified.

At present, the research on the preparation process and microscopic mechanism coatings for electromagnetic rail launch is still in its infancy, especially in mechanical and thermal shock tests of surface coatings to simulate electromagnetic launch processes. Among various kinds of coating preparation technologies, laser cladding technology has the advantages of high flexibility, small thermal influence on the workpiece, and high bonding strength between the coating and the substrate. A fine-grained, high-performance coating can be obtained with this technique. Thus, laser cladding technology is a promising surface treatment technology for the A&R.

### 3.3. Summary of Protection Strategies for Surface Damage

Much progress has been made in the material improvement and surface treatment technology for A&R materials for electromagnetic rail launch. Although considerable research has been conducted on the strength and electrical conductivity of rail materials, current copper alloy materials still cannot meet the requirements for continuous long-term applications in special environments, such as extreme mechanical wear and high-temperature thermal shock. While various new wear-, ablation-, and melting-resistant coatings on the surfaces of the A&R can indeed mitigate mechanical, electrical, and partial coupling damage, such as armature melting and deposition, to some extent, the coatings on the surface still pose certain challenges in practical launch systems. On the one hand, the current coating fabrication processes struggle to ensure uniformity over large surface areas, particularly for increasingly long rail surfaces. On the other hand, the coatings on the surface often reduce the electrical conductivity of the A&R surfaces, and the disparities between the coating and substrate materials lead to poor interface bonding, impacting the precision of the launches. With respect to rail grooving, the formation mechanism of grooves is currently in the qualitative research stage and cannot fully explain the process. The relationship between groove damage and material characteristics, as well as whether surface treatment techniques such as coatings can effectively suppress groove formation, remains unclear. Further research is required to address the issue of groove protection.

## 4. Conclusions and Outlook

This study examined different damage forms in A&R materials, summarized their damage mechanisms, and analyzed protection strategies for these damages. Although structural improvements are possible, addressing material issues is fundamental for achieving long service life and efficiency. Existing rail materials require further enhancement in terms of their strength and wear resistance without compromising their conductivity. Coating technology can effectively mitigate the damage to A&R materials, but current coating techniques still face challenges, such as the uniform preparation of large-area coatings, imbalances in coating conductivity, wear resistance, and interface bonding properties with A&R materials.

Considering the increasing demand for a higher performance in electromagnetic railgun launch, the main research and development prospects of A&R materials are as follows:

(1) In terms of material, more severe environments result in higher requirements for the A&R materials. High strength and conductivity and wear resistance are the goals of A&R materials. The research on new material systems and combined treatment technology are the direction of the future development of A&R materials.
(2) In terms of coating and preparation technology, large-area uniform coating preparation technology and new materials with high conductivity, wear resistance, ablative resistance, and strong binding forces should be explored to further optimize the quality of the coating.
(3) In terms of the friction pair system, most current research has been aimed at separating the armature from the rail, and different pairing mechanisms should be considered during material development.
(4) In terms of material testing, due to the relatively large cost of electromagnetic emissions, it is necessary to develop material-equivalent test methods to evaluate whether new materials meet actual emission requirements.
(5) In terms of the mechanism of wear and ablation, the understanding of conductive wear resistance and ablation resistance should be deepened. Research on the mechanism of current-carrying tribology and the arc ablation under extreme conditions is highly required.

## Figures and Tables

**Table 1:** Mechanical properties and electrical conductivity of CuCrZr alloys subjected to different processes.

Processing	σ_UTS_ (MPa)	Cond. (%IACS)	Ref.
Aging + cryorolling	712	70.2	[75]
Two-step cryorolling + aging	648	79.8	[76]
Annealing + LNT-DPD ^a^	700	98.5	[77]
ST ^b^ + ECAP ^c^ × 2 + HE ^d^ + aging	625	78.0	[78]
ST ^b^ + HE ^d^ + aging	630	79.0	[78]
ST + CEF ^e^ + drawing + aging	590	77.6	[79]
ST + cold drawing + aging	550	78.7	[79]
ST + ECAP × 4 + aging	577	78.5	[80]

^a^ Dynamic plastic deformation at liquid-nitrogen temperature. ^b^ Solution treatment. ^c^ Equal-channel angular pressing. ^d^ Hydrostatic extrusion. ^e^ Continuous extrusion formation process.

## Data Availability

Data are contained within the article.
